# Microbiome analysis in women with endometriosis: Does a microbiome exist in peritoneal fluid and ovarian cystic fluid?

**DOI:** 10.1002/rmb2.12441

**Published:** 2022-01-29

**Authors:** Sugiko Oishi, Keiko Mekaru, Suguru E. Tanaka, Wataru Arai, Kyota Ashikawa, Yoshiyuki Sakuraba, Mikiko Nishioka, Rie Nakamura, Maho Miyagi, Kozue Akamine, Yoichi Aoki

**Affiliations:** ^1^ Department of Obstetrics and Gynecology Graduate School of Medical Science University of the Ryukyus Okinawa Japan; ^2^ Varinos, Inc. Tokyo Japan; ^3^ Department of Obstetrics and Gynecology Mie University Mie Japan

**Keywords:** 16S rRNA, endometriosis, microbiome, ovarian cystic fluid, peritoneal fluid

## Abstract

**Purpose:**

To investigate the relationship between the microbiome of the female genital tract and endometriosis.

**Methods:**

This prospective cohort study included 36 women who underwent laparoscopic surgery for ovarian tumor from July 2019 to April 2020. Of them, 18 had endometriosis, and 18 did not have endometriosis. Vaginal secretions, endometrial fluid, peritoneal fluid, and ovarian cystic fluid were collected during surgery. Next‐generation sequencing of bacterial 16S rRNA was performed to characterize the microbiome.

**Results:**

Specific microbiomes were not detected in either peritoneal fluid or ovarian cystic fluid regardless of the presence or absence of endometriosis and the type of cyst. When the cutoff value of infectious bacterial abundance in the vagina was set as 64.3%, there were many cases more than a cutoff value in the endometriosis group significantly (*p* = 0.01). When the cutoff value of infectious bacterial abundance in the endometrium was set as 18.6%, there were many cases more than a cutoff level in the endometriosis cases significantly (*p* = 0.02).

**Conclusion:**

Peritoneal fluid and ovarian cystic fluid are almost sterile, although dysbiosis may occur in the vaginal and endometrial microbiome in women with endometriosis.

## 1 INTRODUCTION

Endometriosis is a disease that occurs outside the endometrial cavity. Endometrial tissue and similar tissues develop into various lesions, such as the peritoneal cavity and inside the ovary. It sometimes causes adhesion lesions in the abdominal cavity; therefore, the main symptom is abdominal pain during menstruation. Although the etiology is unclear, endometriosis is one of the most common diseases in women of reproductive age. An increasing prevalence of endometriosis has been reported in approximately 11% of women of reproductive age, which could be attributed to lifestyle changes.^1^ Therefore, endometriosis can considerably affect womens’ quality of life, leading to a deterioration of not only their personal life but also social and/or professional life, considering the modern and advanced lifestyle of women at this age.^2^ It is common for women with endometriosis to experience iatrogenic pelvic inflammatory disease and the development of a tubo‐ovarian abscess following endometrium biopsy, hysterosalpingography, and oocyte retrieval.^3^ These cases may follow iterative and intractable progression and often are difficult to treat. Additionally, endometriosis is a cause of sterility because of implantation failure.^4^

The association between endometriosis and infection has been assessed, and recently, some studies have reported the microbiome of the female genital tract.^5^, ^6^ According to these reports, the microbiome is affected by age, reproductive condition, ethnicity, and other factors, as well as by highly dynamic changes throughout life. Some microorganisms may increase the risk of genital tract infection.^7^ Therefore, we assumed that a microbiome exists in patients with endometriosis, which we could not detect using normal bacteriological culture methods, and that it is related to chronic inflammatory conditions with endometriosis.

Recently, a small number of bacteria were noted to be present in the peritoneal fluid (PF) and endometrium, which was thought to be sterile.^8^, ^9^ However, it is unclear how the microbiome of the PF and endometrium affects human health. Because of the difficulty in measuring small numbers of microorganisms, the abdominal cavity and uterus were thought to be sterile. Using 16S rRNA sequence analysis, we amplified the 16S rRNA domain of bacteria and analyzed the microbiome with a little sample more precisely by analysis of sequence arrangement using next‐generation sequencing in large quantities. Thus, we identified the bacteria present and characterized the bacterial community without culturing.^10^

In this study, we clarified the relationship between the microbiome of the female genital tract and endometriosis by confirming the existence of the microbiome in the vagina, endometrium, PF, and cystic fluid in women with endometriosis.

## 2 MATERIALS AND METHOD

### 2.1 Study design/patients/purpose

This was a prospective cohort study of 36 women with or without endometriosis who had ovarian tumors and underwent laparoscopic surgery at Ryukyu University Hospital and Mie University Hospital from July 2019 to April 2020. The inclusion criteria were patients aged ≥20 years who provided consent with a preoperative diagnosis of unilateral or bilateral benign ovarian tumors. Among them, 18 patients had endometriosis (Endo group), and 18 did not have endometriosis (Non‐Endo group). We excluded postmenopausal patients, patients with uterine anomalies, and patients who used antibiotics. Patients without endometriosis who were found to have endometriosis lesions in the abdominal cavity at surgery were also excluded. The outcome was to evaluate differences in the microbiome of the PF, ovarian cystic fluid, endometrium, and vagina in patients with and without endometriosis.

This study complied with the principles of the Declaration of Helsinki (October 2013 correction) and “ethic guidelines about the medical system study for people.” We obtained consent from all patients. The Institutional Review Board (IRB) of the University of the Ryukyus, Mie University, and Varinos Inc. approved this study (IRB No. 1369).

### 2.2 Sample collection

We collected vaginal secretions (VS), endometrial fluid (EF), PF, and ovarian cystic fluid (OF) of patients with endometriosis and without endometriosis during surgery. VS samples were collected by swab before vaginal sterilization. After collecting VS samples, we sterilized the vagina with povidone‐iodine three times, washed it with saline, and collected EF using a brush for cell collection (ASKA Pharmaceutical, Tokyo, Japan). PF samples were collected by suction using a sterile procedure during laparoscopic surgery. In a case where none of the PF were recognized, we washed the abdominal cavity with 15–20 ml saline and collected the sample. We collected OF samples by puncturing the ovarian tumor directly in a bag aseptically after salpingo‐oophorectomy or via the abdominal wall using a sterile procedure during cystectomy.

### 2.3 Microbiome analysis

The hypervariable regions of the variable regions 1–2 (V1–2) of the bacterial 16S rRNA gene were amplified and analyzed using next‐generation sequencing to identify the bacteria.

#### 2.3.1 DNA extraction, sequencing, and analysis of sequencing data

The VS, EF, PF, and OF samples were treated with proteinase K (Roche Applied Science, Penzberg, Germany) containing 100 mg/ml lysozyme solution (Sigma‐Aldrich, MO, USA).

Genomic DNA was extracted using a MagNA Pure 24 (Roche Diagnostics, Grenzach‐Wyhlen, Germany) Pathogen 1000 hp 3.1 protocol. Ultra‐low biomass samples of the endometrium are greatly affected by reagents and bacteria derived from the working environment. Therefore, an experiment was conducted using ultrapure water as a negative control, and bacteria either derived from reagents or the surrounding environment were monitored. After amplifying the V1–2 of the bacterial 16S rRNA gene, the final library was paired‐end sequenced at 2 × 251 bp using a MiSeq Reagent Kit v3 on the Illumina MiSeq platform (Illumina, Inc., San Diego, CA, USA). Operational taxonomic units (OTUs) were constructed after quality filtering of the paired‐end reads. The OTUs were assigned to taxa using the database reported in a previous study.^11^ Bacteria that were frequently observed in the negative control were grouped as background bacterial contamination (Supplemental Data 1), and after screening of the samples, the background‐contaminated bacteria were excluded from the microbiome profile.

#### 2.3.2 Sample screening and clustering

Nonhierarchical clustering of microbiome profiles in VS, EF, PF, and OF samples and negative controls were conducted using weighted UniFrac distance. Samples clustered with negative controls were not used for subsequent analyses. Hierarchical clustering of VS and EF samples with microbiome profiles excluding background‐contaminated bacteria was conducted using Bray‐Curtis distance matrix, and heatmaps were generated.

After rarefaction analysis, including Shannon index, Chao1 richness, and PD whole tree using microbiome profiles, alpha diversity indexes were compared between the Endo and Non‐Endo groups in VS and EF samples. Beta diversity was analyzed using principal coordinate analysis. Multivariate analysis based on weighted UniFrac distance was conducted to compare differences in the microbiome between the Endo and Non‐Endo groups in VS and EF samples. Beta diversity was analyzed using permutational multivariate analysis of variance (PERMANOVA) test.

Infectious bacteria were defined by previous studies (Table 1).^1^, ^12^, ^13^, ^14^, ^15^, ^16^, ^17^, ^18^, ^19^, ^20^, ^21^, ^22^ We defined the sum of these infectious bacteria as Infect MB and a combination of *Lactobacillus* and *Bifidobacterium* species as Lactic MB. The abundance of *Lactobacillus*, *Bifidobacterium*, and infectious bacteria were compared between the Endo and Non‐Endo groups in VS and EF samples.

**TABLE 1:** Infectious bacteria

*Aerococcus christensenii*	*Gardnerella vaginalis*	*Prevotella bivia*	*Streptococcus mitis*
*Atopobium vaginae*	*Haemophilus parainfluenzae*	*Prevotella buccalis*	*Streptococcus oralis*
*Bacteroides fragilis*	*Unclassified Megasphaera*	*Prevotella disiens*	*Streptococcus pneumoniae*
*Bacteroides uniformis*	*Unclassified Mobiluncus*	*Prevotella intermedia*	*Streptococcus pseudopneumoniae*
*Corynebacterium riegelii*	*Mobiluncus mulieris*	*Prevotella oris*	*Streptococcus salivarius*
*Enterococcus faecalis*	*Parabacteroides merdae*	*Prevotella timonensis*	*Streptococcus sanguinis*
*Escherichia coli*	*Peptoniphilus harei*	*Sneathia amnii*	*Streptococcus vestibularis*
*Unclassified Fastidiosipila*	*Peptoniphilus lacrimalis*	*Streptococcus agalactiae*	*Unclassified Ureaplasma*
*Finegoldia magna*	*Peptostreptococcus anaerobius*	*Streptococcus anginosus*	*Ureaplasma urealyticum*
*Fusobacterium nucleatum*	*Porphyromonas uenonis*	*Streptococcus gordonii*	

Note

Infectious bacteria (Infect MB) were defined by previous studies.^1^, ^12^, ^13^, ^14^, ^15^, ^16^, ^17^, ^18^, ^19^, ^20^, ^21^

The relationship of the rate of Lactic MB in VS and EF samples with endometriosis was analyzed using receiver operating characteristic (ROC) analysis, and the best criteria of the combination rate were investigated. ROC analysis was also conducted for Infect MB using the abovementioned method.

### 2.4 Statistical analysis

All analyses were conducted using R software version 3.6.2. The normality and homoscedasticity of continuous data were analyzed using the Shapiro‐Wilk test and Bartlett's test, respectively. When the data had normality and homoscedasticity, Student's *t* test was used. When the data had only normality, Welch's *t* test was used. When the data did not have normality, Wilcoxon's rank‐sum test was used. For discrete data, Fisher's exact test was used. A *p*‐value of <0.05 was considered statistically significant.

## 3 RESULTS

### 3.1 Background

The patients' backgrounds are shown in Table 2. The mean ages of the Endo and Non‐Endo groups were 37.9 and 35.2 years, respectively, and no significant difference was noted (*p* = 0.29). Bilateral ovarian lesions (*n* = 10) were more common than unilateral lesions (*n* = 8) in the Endo group compared with that in the Non‐Endo group, in which only one patient had bilateral ovarian lesions. Five cases were stage Ⅲ and 13 cases were stage IV based on the revised American Society for Reproductive Medicine (rASRM) score in the Endo group. The median rASRM score was 66. Various types of ovarian tumors were noted in the Non‐Endo group. The most frequent type of tumor was mature cystic teratoma (*n* = 14). Among these patients, one patient was identified to have mixed carcinoid components by pathology after surgery. Regarding other patients, mucinous cyst adenoma (*n* = 2), paraovarian cyst (*n* = 1), and struma ovarii (*n* = 1) were noted. There were no surgical findings indicative of early signs of endometriosis in the Non‐Endo group.

**TABLE 2:** Characteristics of Endo and non‐Endo groups

	Endo group	Non‐Endo group		*p*‐value
	(*n* = 18)	(*n* = 18)		
Age (years, mean ± SD)	37.9 ± 6.4	35.2 ± 8.6		0.29
Range in age (years)	27–49	20–49		
Parity (*n*, mean ± SD)	0.6 ± 0.8	1.1 ± 1.3		0.29
Gravidity (*n*, mean ± SD)	0.4 ± 0.8	0.8 ± 1.0		0.18
Body mass index	22.4 ± 2.9	23.0 ± 4.9		0.99
Type of tumor	Endometriosis	Mature cystic teratoma	*n* = 14	
		Mucinous cyst adenoma	*n* = 2	
		Paraovarian cyst	*n* = 1	
		Struma ovarii	*n* = 1	
Site of lesion (*n*)				
Bilateral	10	1		
Unilateral	8	17		
Maximum diameter of tumor (cm, mean ± SD)	6.2 ± 2.8	7.7 ± 3.0		0.17
Revised ASRM staging				
Stage Ⅲ	5			
Stage IV	13			
Hormone use within 3 months of surgery (*n*)	4	1		0.34

### 3.2 The microbiome of PF and OF

We found 121 and 94 species in PF and OF, respectively. However, almost all PF and OF samples were clustered, similar to negative controls, and the correlation coefficient between PF samples and the negative control was approximately “1.00.” In addition, the number of sequence reads after filtering was extremely low. Therefore, we concluded that almost all PF and OF samples had no specific microbiome both in the Endo and Non‐Endo groups. Thus, the PF and OF samples were excluded from subsequent analyses.

We could not detect any specific microbiome in PF and OF, though we detected very small numbers of bacteria, such as *Paracoccus yeei*, which were not present in the negative control. This implies that the possible existence of small quantities of distinctive bacteria cannot be denied.

### 3.3 The microbiome of the vagina and endometrium

**FIGURE 1:**
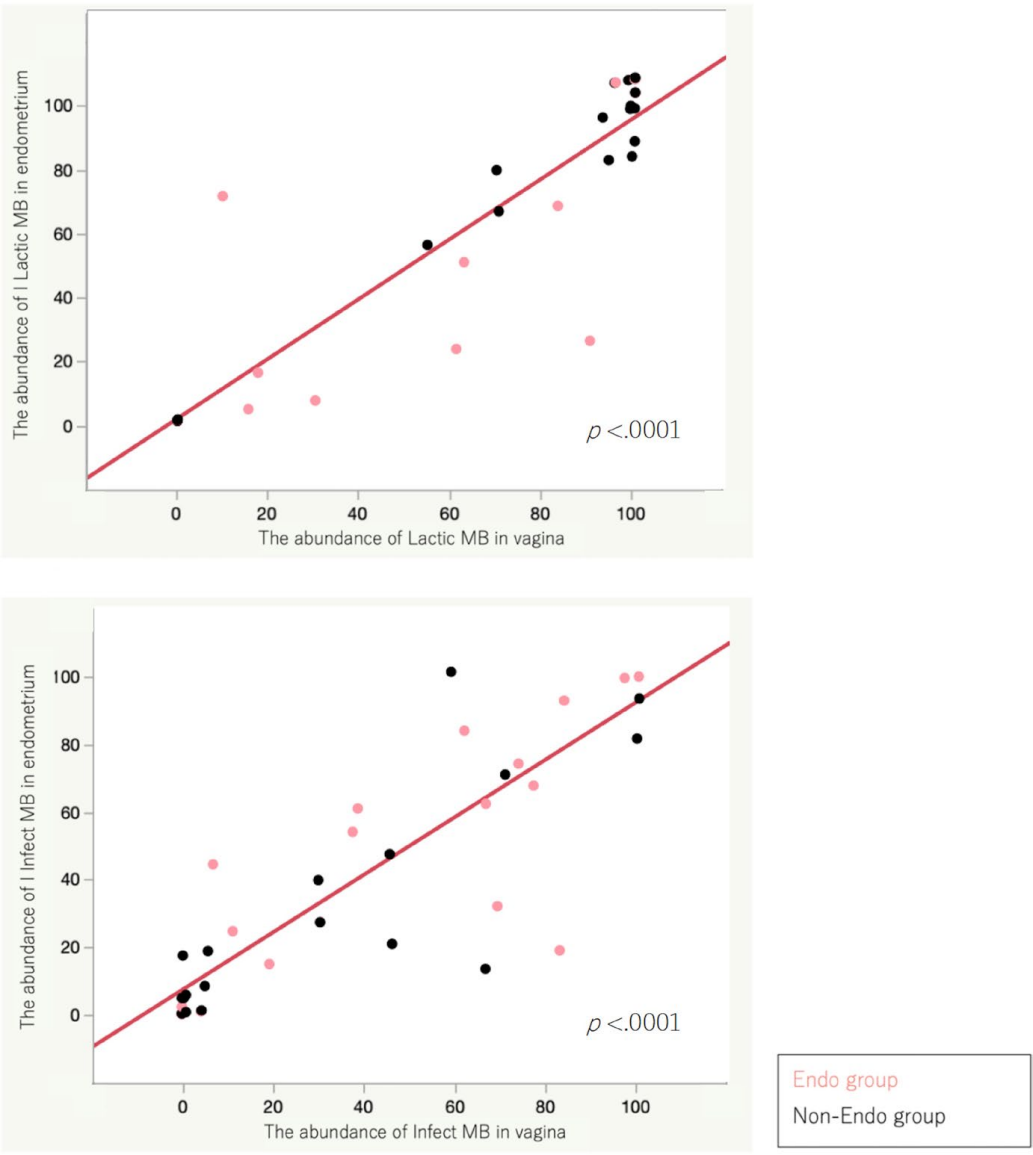
Correlation of Lactic and Infect MBs in vagina and endometrium. There was a strong correlation between vaginal and endometrial microbiome in the abundance of Lactic and Infect MBs (*p* < 0.0001)

**FIGURE 2:**
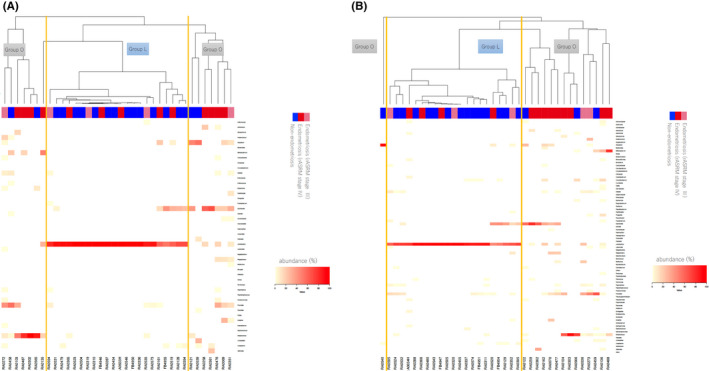
Clustering analysis. (A) The clustering analysis of vagina at the genus level. Group L = Group which the highest abundance of bacteria was *Lactobacillus*. Group O = Group which the highest abundance of bacteria was except *Lactobacillus*. There were significantly many endometriosis cases in Group O in the vaginal microbiome (*p* = 0.04). (B) The clustering analysis of endometrium at the genus level. There were significantly many endometriosis cases in Group O in the endometrial microbiome (*p* = 0.02)

We found 120 and 151 species in the vagina and endometrium, respectively. The microbiome of the vagina and endometrium were similar. The abundance of Lactic and Infect MBs in both the vagina and endometrium was correlated (Figure 1). The cluster analysis of the microbiome of the vagina and endometrium at the genus level is shown in Figure 2.

Two groups were detected in the cluster analysis of the microbiome of the vagina and endometrium; one group had the highest abundance of *Lactobacillus* (Group L) and the other group had a low abundance of *Lactobacillus* (Group O). There were significantly many endometriosis cases in Group O in both vaginal and endometrial microbiome (Figure 2). Differences in the severity of endometriosis were not apparent. The significant difference was seen in Shannon index between the Endo and Non‐Endo groups in both the vagina and endometrium, although was not seen in Chao 1 richness and PD whole tree (Supplemental Data 2). There was different clustering of microbiome in the vagina and endometrium between the Endo and Non‐Endo groups as determined using beta diversity analysis by PCoA plots (Supplemental Data 3).

The abundance of Lactic MB in the vagina and endometrium did not differ between the Endo and Non‐Endo groups and was the same as that of Infect MB (Supplemental Data 4). Therefore, we established a cutoff value of the abundance of Lactic and Infect MBs in the vagina and endometrium using ROC analysis to analyze the difference between the Endo and Non‐Endo groups (Supplemental Data 5).

**FIGURE 3:**
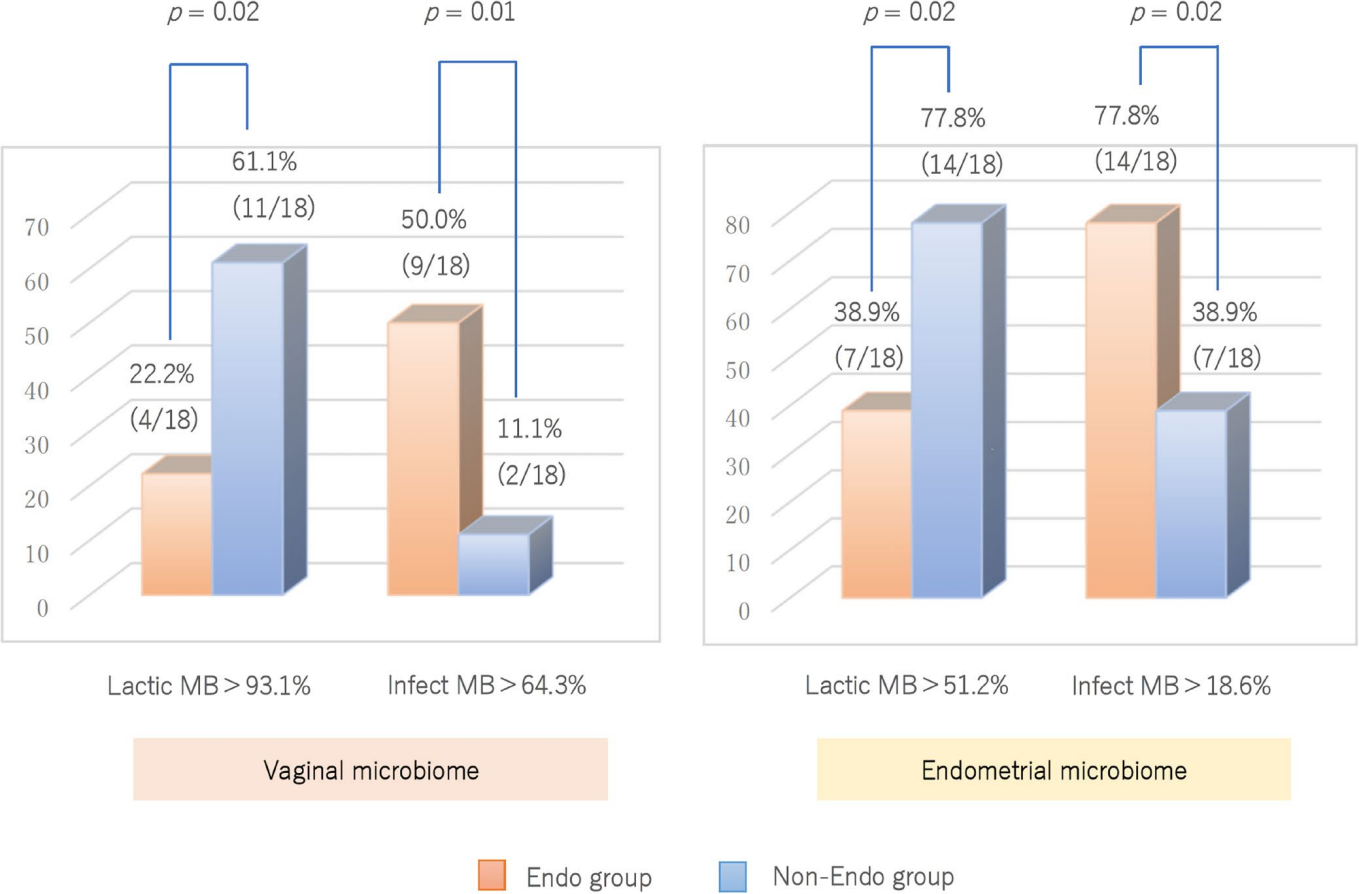
Analysis of Lactic and Infect MBs in vagina and endometrium using cutoff value by ROC curve. There were significantly few cases with Lactic MB more than cutoff levels and were many cases with Infect MB more than cutoff levels in Endo Group at the vagina and endometrium

When we set the cutoff value of the abundance of vaginal Infect MB as 64.3%, there were many cases more than a cutoff level in the Endo group significantly (50.0%, 9/18 vs. 11.1%, 2/18; *p* = 0.01). In contrast, when the cutoff value of vaginal Lactic MB abundance was set to 93.1%, the Endo group have significantly fewer cases below the cutoff level (22.2%, 4/18 vs. 61.1%, 11/18; *p* = 0.02) (Figure 3).

When we set the cutoff value of endometrial Lactic MB abundance to 51.2%, there were many cases less than a cutoff level in the Endo group significantly (38.9%, 7/18 vs. 77.8%, 14/18; *p* = 0.02). When the cutoff value of endometrial Infect MB abundance was set as 18.6%, there were many cases more than a cutoff level in the Endo group significantly (77.8%, 14/18 vs. 38.9%, 7/18; *p* = 0.02).

There were significantly few cases with Lactic MB more than cutoff levels and were significantly many cases with Infect MB more than cutoff levels at the vagina and endometrium in the Endo group.

## 4 DISCUSSION

In this study, PF and OF appear to be sterile regardless of the possible presence of endometriosis and the type of cyst, although we were able to show that dysbiosis may occur in women with endometriosis in the vaginal and endometrial microbiome.

The etiology of endometriosis is unclear, although various methods of onset have been hypothesized. One hypothesis, “implantation of endometrium,” states that the endometrium refluxes into the peritoneal cavity with menstrual blood from the fallopian tubes.^23^ According to this hypothesis, the risk factor of onset is said to be related to the presence of inflammation. The mechanism of onset of chronic pelvic inflammation is unknown, although some recent reports suggest a relationship with the microbiome. Khan et al. reported that the lower genital tract in humans is constantly exposed to microorganisms, which could infect the upper genital tract through direct migration. They suggested that bacterial infection after migration from the vagina to contaminating menstrual blood results in the accumulation of endotoxin in the PF and initiation of pelvic inflammation.^24^ Additionally, they reported that the levels of cytokines or growth factors increase in the PF of patients with endometriosis, which may lead to the progression of endometriosis lesions.^24^

Although it was thought that the abdominal cavity was sterile, a microbiome was found to be present in PF.^25^ The microbiome of the endometriotic cyst was also supposed to be present, and two courses of migration, that is, the ascending course from the vagina and from the bowels via adhesion in the abdominal cavity, were assumed to take place.^26^ A recent review of the microbiome in patients with endometriosis indicated that species belonging to the phylum *Proteobacteria*, which increased during an inflammatory state, were significantly increased in the endometrium, PF, and endometriotic cyst in patients with endometriosis.^27^

Because it was regarded that the microbiome is formed by microorganisms that ascend from the vagina, we sought to prove the ascent of bacteria by analyzing a series of microbiomes of the vagina, endometrium, PF, and OF simultaneously because a common pattern was estimated to be recognized. However, we could not detect any meaningful microbiome in PF and OF. Although some studies have detected microbiomes in PF,^8^, ^26^ differences were noted between these previous studies and the present study. This was a result of comparing the microbiome of samples with the negative control and removing contamination of an extremely small number of bacteria in the surrounding environment. On the other hand, the original microbiome was seen in the vagina and endometrium, and there were many cases with a high abundance rate of bacteria relating to infection in the Endo group in both the vagina and endometrium. In our study, we analyzed infectious bacteria by grouping as Infect MB across the distinction between phyla. Thus, it is a new viewpoint which is not seen before. Because the outcome was to evaluate differences in the microbiome between cases with and without endometriosis, we could not use the existing cutoff values for pregnancy outcome.^9^, ^28^ Therefore, we thought it was necessary to establish a new cutoff value as an indicator to be applied in clinical practice and to determine the presence of endometriosis. In this analysis, we tested multiple cutoff values and set the value with the lowest *p*‐value and highest accuracy as the cutoff value (Supplemental Data 5). It is well understood that *Lactobacillus* produces lactic acid and hydrogen peroxide to prevent inflammation, and *Bifidobacterium* produces acetic acid and aggravates the barrier function of the mucous membrane.^29^ We found a low abundance of bacteria working with such mechanisms in the Endo group with tendency in both the vagina and endometrium. However, increased abundance of *Bifidobacterium* has been reported in mouse models of endometriosis,^30^ and thus far, the presence of endometriosis is not necessarily associated with a decrease in the abundance of bacteria such as *Lactobacillus* and *Bifidobacterium*.

The association between bacterial inflammation and endometriosis has been reported until now,^24^ although it is unknown whether bacterial infection develops endometriosis or endometriosis resulted in bacterial infection by an immunologic abnormality or other reasons. García et al.^31^ hypothesized that there might be a direct relationship between higher prenatal exposure to endocrine‐disrupting chemicals and a higher risk of developing endometriosis in adulthood. They hypothesized that a high level of endocrine‐disrupting chemicals during the prenatal stage induces a shorter anogenital distance that could produce dysbiosis of the vagina, which supports subclinical inflammation related to *Gardnerella*, *Prevotella*, *Mobiluncus*, *Sneathia* possibly develops endometriosis.^31^ In recent studies, it was discovered that microbiome directly contributes to the host's immunoresponse.^32^ Bacterial species such as *Citrobacter rodentium* and *Escherichia coli* O‐157 may be inducing Th17 cell, which causes inflammation from the CD4‐positive T cell of the host after gluing to the small intestine epithelium.^32^ Furthermore, *Bacteroides*, which is found in the endometrium, as shown by 16S rRNA sequence analysis, modulates the Th17 response of intestinal T cells and causes a systemic increase in circulating CD4+ T cells and Th1 cells.^33^ In addition, some studies reported there were abundant inflammatory cells, cytokines, and growth factors in the abdominal cavity of women with endometriosis, and various cytokines were produced by endometriotic stromal cells, which was an endometriotic lesion itself, and had a relationship with a self‐increase and pathologic progress.^34^ These studies suggest that bacterial infection may be involved in the development and progression of endometriosis. In this study, there was a tendency to cluster the direction abounding in *Gardnerella* in the Endo group and *Lactobacillus* in the Non‐Endo group in both the vagina and endometrium according to clustering analysis and beta diversity analysis. Thus, dysbiosis of the microbiome of the vagina and endometrium in the Endo group may occur, and it may be connected with the onset and progress of endometriosis by a mechanism, such as in a previous study.

However, it is suggested that the survival of endometriotic tissues is permitted because of an abnormal immunoresponse, such as a decrease in surveillance to remove an ectopic endometrium in women with endometriosis.^35^, ^36^ Therefore, we cannot exclude the possibility that bacterial infection develops due to immunological abnormality. Furthermore, evidence that endometrial bacteria induce host immunity in the same way as intestinal bacteria has not yet been established. Thomas‐White et al. speculated that intestinal bacteria and bacteria in the reproductive system possess different functions.^37^ Detailed genomic and functional comparison of the urogenital microbiome with the gastrointestinal microbiome demonstrated urogenital functional capacities distinct from those observed in the gastrointestinal microbiome.^37^ Therefore, further research is warranted to investigate the microbiome and human immunity in relation to endometriosis.

Many patients with endometriosis are annoyed by infertility, and one of the factors that contribute to sterility is implantation disorder. A potential reason for implantation disorder is the decreased expression of biochemical markers of decidualization in the endometrium under the influence of increasing cytokine levels in endometriotic PF^38^ and the presence of chronic endometritis.^39^ Khan et al.^40^ reported the possibility of allowing the lesion of chronic endometritis and endometriosis regress with the improvement of dysbiosis using broad‐spectrum antimicrobials in women with endometriosis. This is expected to improve infertility outcomes by ameliorating dysbiosis. Further investigation will be needed to determine which treatments improve clinical outcomes such as infertility and pain of the endometriosis by correcting dysbiosis.

To the best of our knowledge, this is the first study to simultaneously analyze the microbiome of the vagina, endometrium, PF, and OF. The limitations of this study include the small number of patients and lack of information about previous treatment of bacterial vaginosis. The existence of cases with hormone therapy before surgery and the lack of information on the menstrual cycle that may affect the microbiome are also serious limitations. We found that PF and OF were approximately sterile regardless of the presence of endometriosis and the type of cyst, implying that we were able to eliminate bacterial contamination in the sample collection and inspection process. This study suggests that dysbiosis may occur in women with endometriosis because there were many patients with a high abundance of infectious microorganisms and fewer patients with a high abundance of *Lactobacillus* and *Bifidobacterium* spp. in the Endo group in both the vagina and endometrium. Further research is needed to clarify whether dysbiosis really exists in endometriosis.

## CONFLICT OF INTEREST

Sugiko Oishi, Keiko Mekaru, Suguru Tanaka, Wataru Arai, Kyota Ashikawa, Mikiko Nishioka, Tadashi Maezawa, Rie Nakamura, Maho Miyagi, Kozue Akamine, and Yoichi Aoki declare that they have no conflict of interest. Yoshiyuki Sakuraba serves as a Chief Executive Officer at Varinos Inc. and has stock ownership of Varinos Inc.

## ETHICAL APPROVAL

This study was approved by the Institutional Review Board (IRB) at the University of the Ryukyus, Mie University, and Varinos Inc. (November 1, 2018; IRB No. 1369).

## HUMAN RIGHTS STATEMENTS AND INFORMED CONSENT

All procedures in this study were conducted in accordance with the ethical standards of the responsible committee on human experimentation (institutional and national) and with the principles of the Helsinki Declaration of 1964 and its later amendments. Informed consent was obtained from all patients. This article does not contain any studies with animal subjects.

## CLINICAL TRIAL REGISTRY

Not applicable.

## Supporting information

Supplementary Material
