# Supplementary material for: Microbiome analysis in women with endometriosis: Does a microbiome exist in peritoneal fluid and ovarian cystic fluid?
Source: Reprod Med Biol. 2022 Jan 29;21(1):e12441. doi: 10.1002/rmb2.12441 (PMC8967307; doi:10.1002/rmb2.12441)
Supplement: Supplementary file 1 — Supplementary Material [file RMB2-21-e12441-s001.docx]

Supporting Information

Supplemental data 1.

Bacteria excluded from analysis as background bacteria frequently observed in negative control

| *Acidovorax delafieldii* | *Afipia broomeae* | *Pandoraea apista* | *Ralstonia pickettii* |
| --- | --- | --- | --- |
| *Acinetobacter bereziniae* | *Brevundimonas diminuta* | *Phyllobacterium myrsinacearum* | *Serratia marcescens* |
| *Acinetobacter guillouiae* | *Cupriavidus metallidurans* | *Pseudomonas extremorientalis* | *Stenotrophomonas maltophilia* |
| *Aeromonas salmonicida* | *Delftia lacustris* | *Pseudomonas migulae* | *Stenotrophomonas pavanii* |
| *Afipia birgiae* | *Delftia tsuruhatensis* | *Pseudomonas rhodesiae* | *Vibrio metschnikovii* |

Supplemental data 2.

The analysis of alpha diversity by the rarefaction analysis in Endo and Non-Endo groups

A. Vaginal microbiome

The significant difference was seen in Shannon index between the Endo and Non-Endo groups in vagina, although was not seen in Chao 1 richness and PD whole tree.

Chao 1 richness

*p* = 0.62

Shannon index

*p* = 0.033

PD Whole Tree

*p* = 0.44


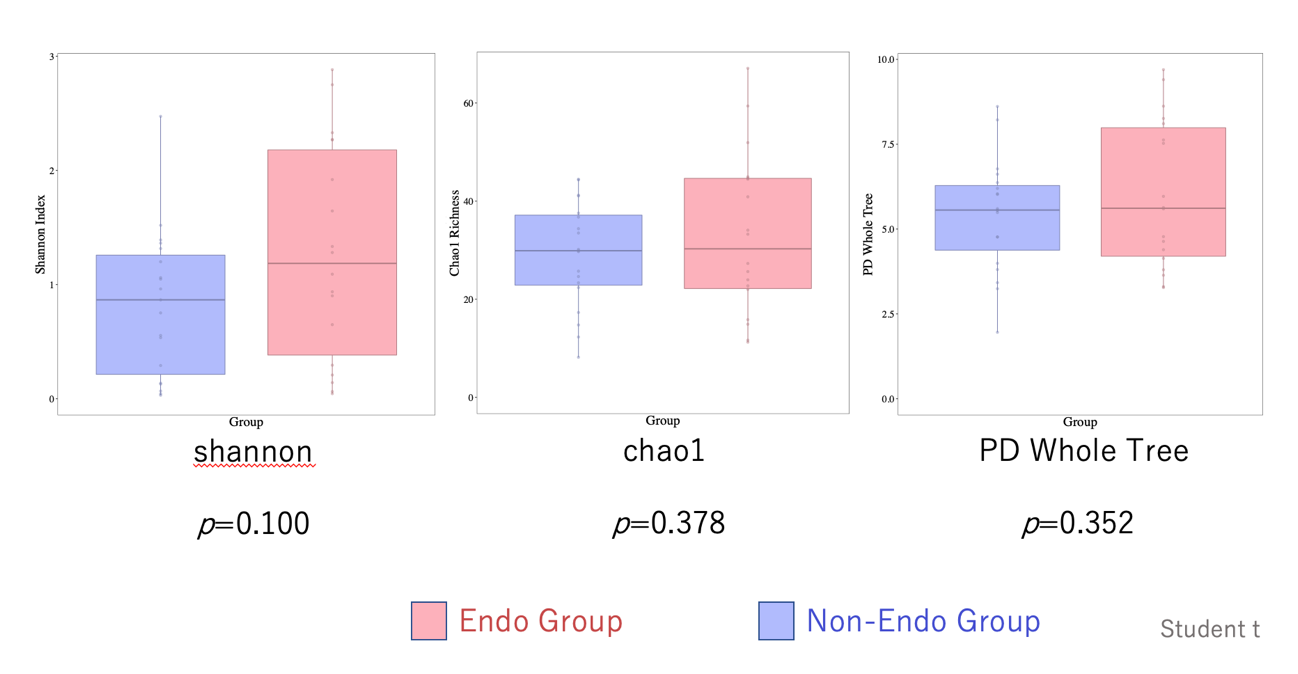


Non-Endo group

Endo group

B. Endometrial microbiome

The significant difference was seen in Shannon index between the Endo and Non-Endo groups in endometrium, although was not seen in Chao 1 richness and PD whole tree.

Chao 1 richness

*p* = 0.53

PD Whole Tree

*p* = 0.86

Shannon index

*p* = 0.029


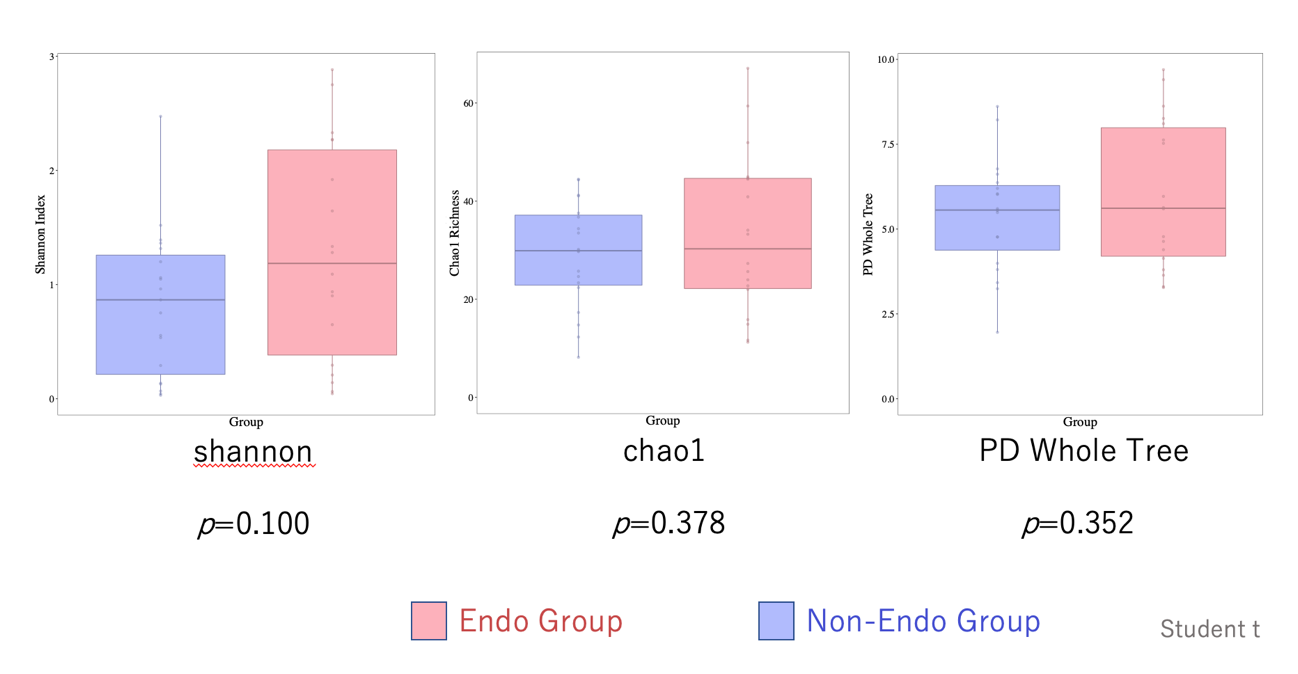


Endo group

Non-Endo group

Supplemental data 3.

The analysis of beta diversity by the princilal coordinate analysis (PCoA) based on weighted UniFrac distance in Endo and Non-Endo groups

1. Vaginal microbiome

There was different clustering of microbiome in the vagina between Endo and Non-Endo groups as observed in PCoA plots (*p* = 0.037).

PERMANOVA test

*p* = 0.037

1. Endometrial microbiome

There was different clustering of microbiome in the endometrium between Endo and Non-Endo groups as observed in PCoA plots (*p* = 0.048).

PERMANOVA test

*p* = 0.048

Supplemental data 4.

The comparison of Lactic and Infect MBs between vaginal and endometrial microbiome.

1. Microbiome of vagina

The abundance of Lactic MB in the vagina did not differ in both Endo and Non-Endo groups, and the same was noted for the abundance of Infect MB

|  | Mean of abundance % ± SD  (Minimum–Maximum) | | *p* value |
| --- | --- | --- | --- |
|  | Endo group  (n = 18) | Non-Endo group  (n = 18) |  |
| Lactic MB | 46.39% ± 42.52  (0.0–100.0) | 72.00% ± 39.04  (0.0–100.0) | 0.062 |
| Infect MB | 47.51% ± 38.17  (0.0–99.9) | 26.95% ± 33.22  (0.0–99.7) | 0.066 |

1. Microbiome of endometrium

The abundance of Lactic MB in the endometrium did not differ in both Endo and Non-Endo groups, and the same was noted for the abundance of Infect MB

|  | Mean of abundance % ± SD  (Minimum–Maximum) | | *p* value |
| --- | --- | --- | --- |
|  | Endo group  (n = 18) | Non-Endo group  (n = 18) |  |
| Lactic MB | 40.42% ± 38.68  (0.0–99.7) | 67.29% ± 37.11  (0.0-99.9) | 0.078 |
| Infect MB | 47.09% ± 36.36  (0.0–98.3) | 27.54% ± 34.08  (0.0–99.7) | 0.207 |

Supplemental data 5. ROC analysis for the presence of endometriosis

Numerics with *p* values < 0.05 are shown in bold.

Lactic MB of vaginal microbiome

| cutoff value | Sensitivity | Specificity | Accuracy | *p* value |
| --- | --- | --- | --- | --- |
| 93.1 | 0.611 | 0.778 | 0.694 | **0.020** |
| 21.4 | 0.833 | 0.500 | 0.667 | **0.038** |
| 94.4 | 0.556 | 0.778 | 0.667 | **0.043** |
| 70.1 | 0.722 | 0.611 | 0.667 | **0.046** |
| 91.1 | 0.611 | 0.722 | 0.667 | **0.046** |

Lactic MB of endometrial microbiome

| cutoff value | Sensitivity | Specificity | Accuracy | *p* value |
| --- | --- | --- | --- | --- |
| 51.2 | 0.778 | 0.611 | 0.694 | **0.020** |
| 73.5 | 0.667 | 0.722 | 0.694 | **0.022** |
| 88.5 | 0.500 | 0.833 | 0.667 | **0.038** |
| 46.5 | 0.778 | 0.556 | 0.667 | **0.043** |
| 61.1 | 0.722 | 0.611 | 0.667 | **0.046** |

Infect MB of vaginal microbiome

| cutoff value | Sensitivity | Specificity | Accuracy | *p* value |
| --- | --- | --- | --- | --- |
| 64.3 | 0.500 | 0.889 | 0.694 | **0.014** |
| 6.5 | 0.778 | 0.611 | 0.694 | **0.020** |
| 75.8 | 0.444 | 0.889 | 0.667 | **0.030** |
| 59.8 | 0.500 | 0.833 | 0.667 | **0.038** |
| 5.6 | 0.778 | 0.556 | 0.667 | **0.043** |

Infect MB of endometrial microbiome

| cutoff value | Sensitivity | Specificity | Accuracy | *p* value |
| --- | --- | --- | --- | --- |
| 18.6 | 0.778 | 0.611 | 0.694 | **0.020** |
| 52.9 | 0.556 | 0.778 | 0.667 | **0.043** |
| 16.7 | 0.778 | 0.556 | 0.667 | **0.043** |
| 19.4 | 0.722 | 0.611 | 0.667 | **0.046** |
| 55.3 | 0.500 | 0.778 | 0.639 | 0.082 |
